# Supplementary material for: Discovery of quantum phases in the Shastry-Sutherland compound SrCu2(BO3)2 under extreme conditions of field and pressure
Source: Nat Commun. 2022 Apr 28;13:2301. doi: 10.1038/s41467-022-30036-w (PMC9050886; doi:10.1038/s41467-022-30036-w)
Supplement: Supplementary file 1 — Supplementary Information [file 41467_2022_30036_MOESM1_ESM.pdf]

Supplementary Information for

Discovery of quantum phases in the Shastry-Sutherland compound $SrCu_2(BO_3)_2$ under extreme conditions of field and pressure

Shi, *et al.*

March 31, 2022

Supplementary Figures

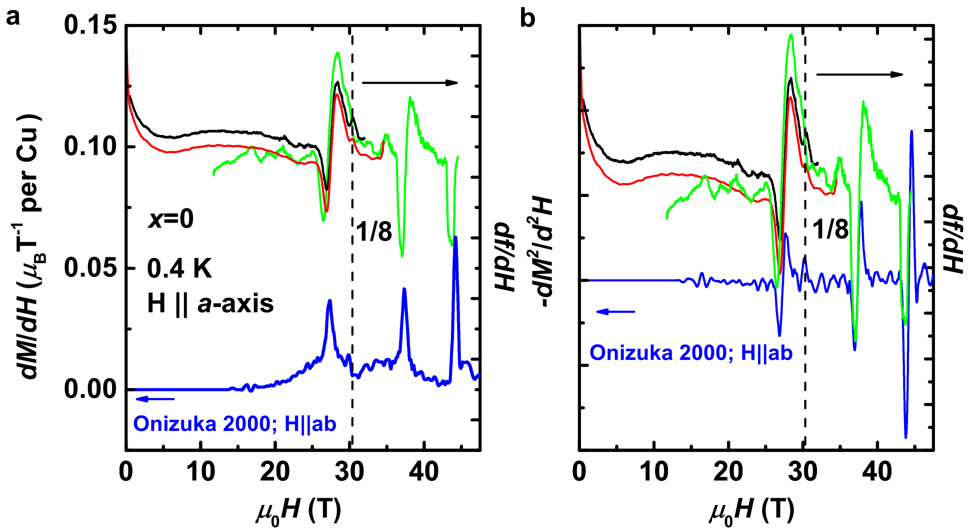

Supplementary Fig. 1: **Identification of the 1/8 plateau at H_1.** (a) dM/dH (left axis) and df/dH (right axis) vs $\mu_0 H$. (b) dM^2/d^2H (left axis) and df/dH (right axis) vs $\mu_0 H$. dM/dH and dM^2/d^2H are calculated based on the magnetization data from Ref. [1]. The same anomaly is clearly seen in both the magnetization measurement [1] and our TDO measurements.

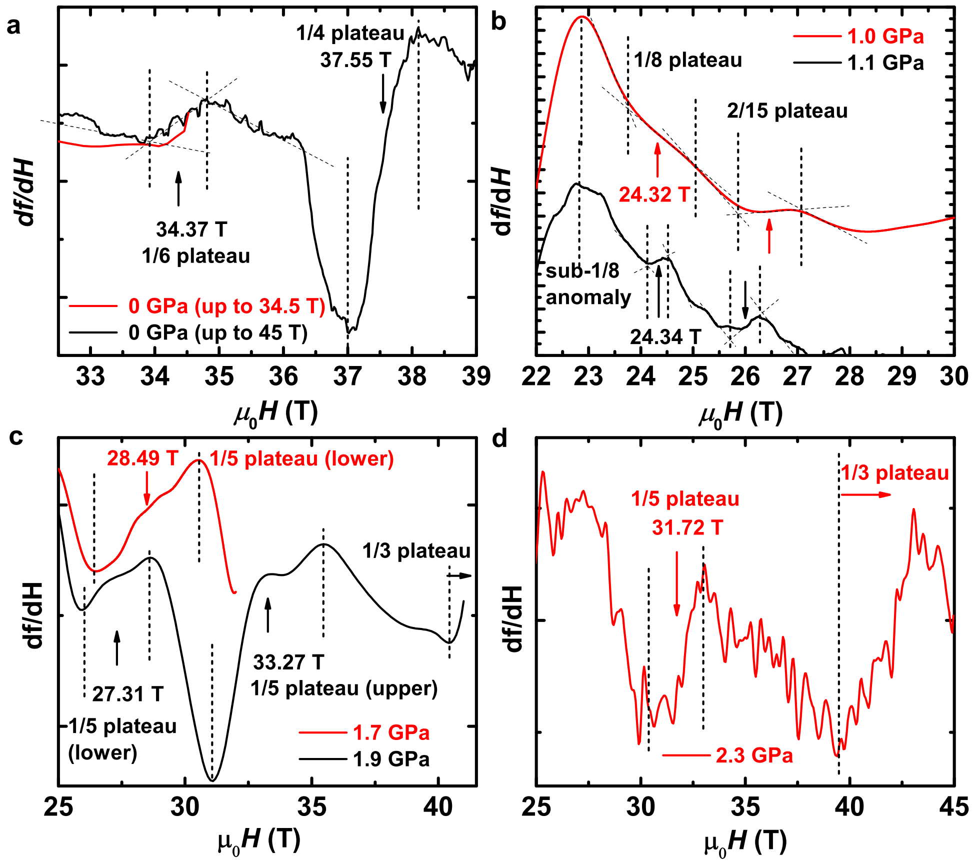

Supplementary Fig. 2: **Identification of the TDO anomalies in** df/dH **vs** $\mu_0 H$ **for the pure sample;** $T = 0.3$ **K.** (**A**) Ambient pressure ($P = 0$); (**B**) $P = 1.0$ GPa and 1.1 GPa; (**C**) $P = 1.7$ GPa and 1.9 GPa; (**D**) $P = 2.3$ GPa. Local minimum ("dip") and local maximum ("bump") are identified at each anomaly, as indicated by the vertical short dashed lines. For weak features, dashed lines are drawn as a guide to eye. The location of the anomaly is identified as the midpoint between the "dips" and "bumps", which correspond to a jump in $M(H)$ (See Fig. Fig. S1 and Ref. [2]). Only data points that are repeatable and show a systematic trend with pressure are considered. For the 1/3 plateau, the "bump" appear above our maximum field range 45 T for all pressures except 2.3 GPa. For consistency, we identify the 1/3 plateau using the H value at the "dips" for all pressure, and we note that it would slightly underestimate the onset field of the 1/3 plateau. This is indeed observed in Fig. 3 and Fig. S1.

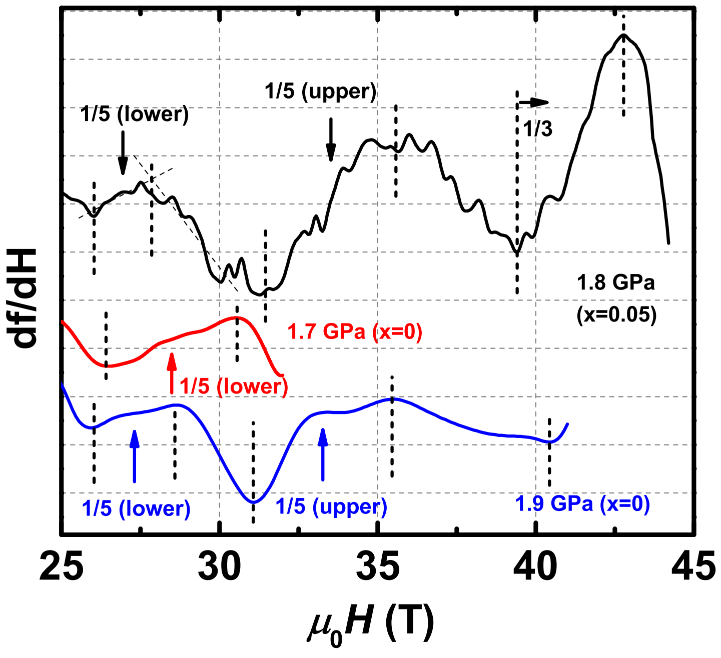

Supplementary Fig. 3: **Identification of the high-P 1/5 and 1/3 plateaus in df/dH vs $\mu_0 H$ for the x=0.05 sample at P = 1.8 GPa and T = 0.3 K.**

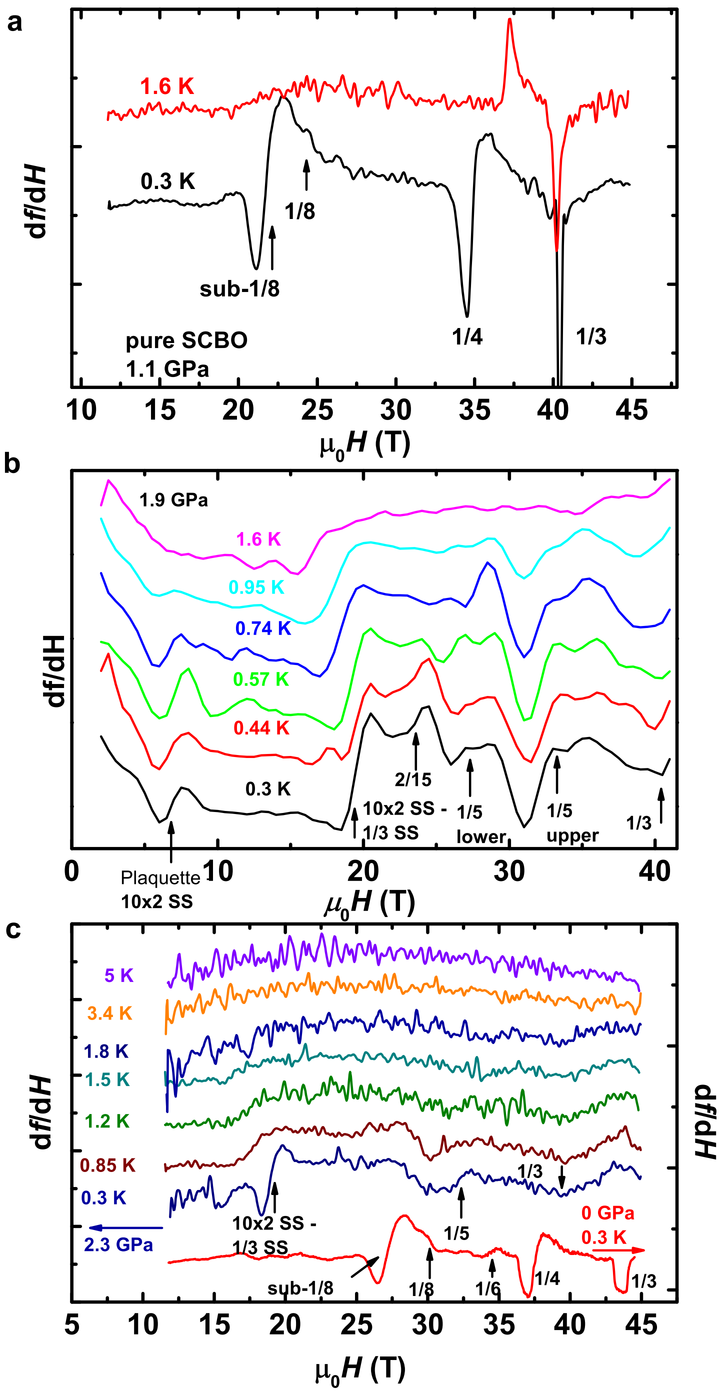

Supplementary Fig. 4: **Temperature dependence of the high-field anomalies.** (a) df/dH vs $\mu_0 H$ at $P = 1.1$ GPa and $T = 0.3$ K and 1.6 K. (b) df/dH vs $\mu_0 H$ at $P =$ 1.9 GPa for T between 0.3 K and 1.6 K. (c) df/dH vs $\mu_0 H$ at $P = 2.3$ GPa (left axis) for various temperatures, in comparison with that at ambient pressure (bottom trace with red color, right axis) and 0.3 K.

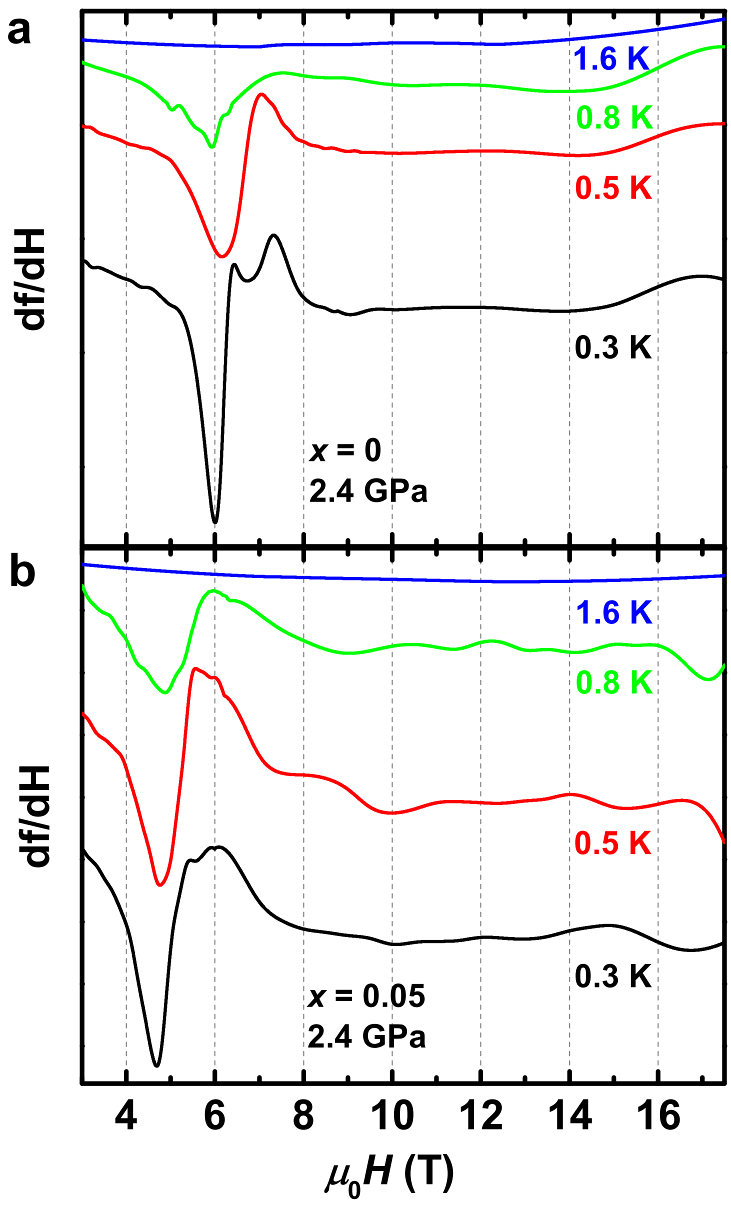

Supplementary Fig. 5: **Doping and temperature dependence of the low-field anomalies at** P **= 2.4 GPa.** df/dH vs $\mu_0 H$ for (a) x=0 and (b) x = 0.05. In both cases, the low-field anomalies are suppressed at 1.6 K. Notably, the low-field anomalies are shifted to lower fields in the x = 0.05 doped sample.

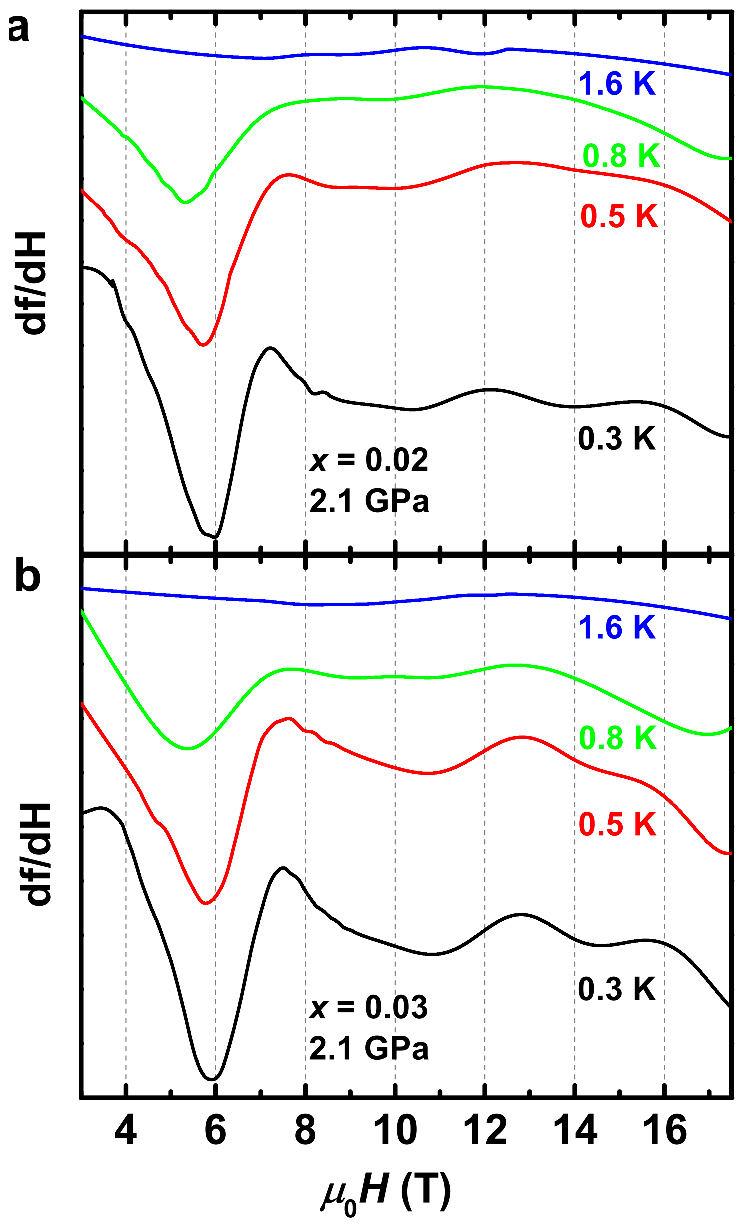

Supplementary Fig. 6: **Doping and temperature dependence of the low-field anomalies at** P **= 2.1 GPa.** df/dH vs $\mu_0 H$ for (a) x=0.02 and (b) x = 0.03. In both cases, the low-field anomalies are suppressed at 1.6 K. Note that the pressure (2.1 GPa) is lower than the one (2.4 GPa) used in Supplementary Fig. 5, so caution is needed when making a comparison.

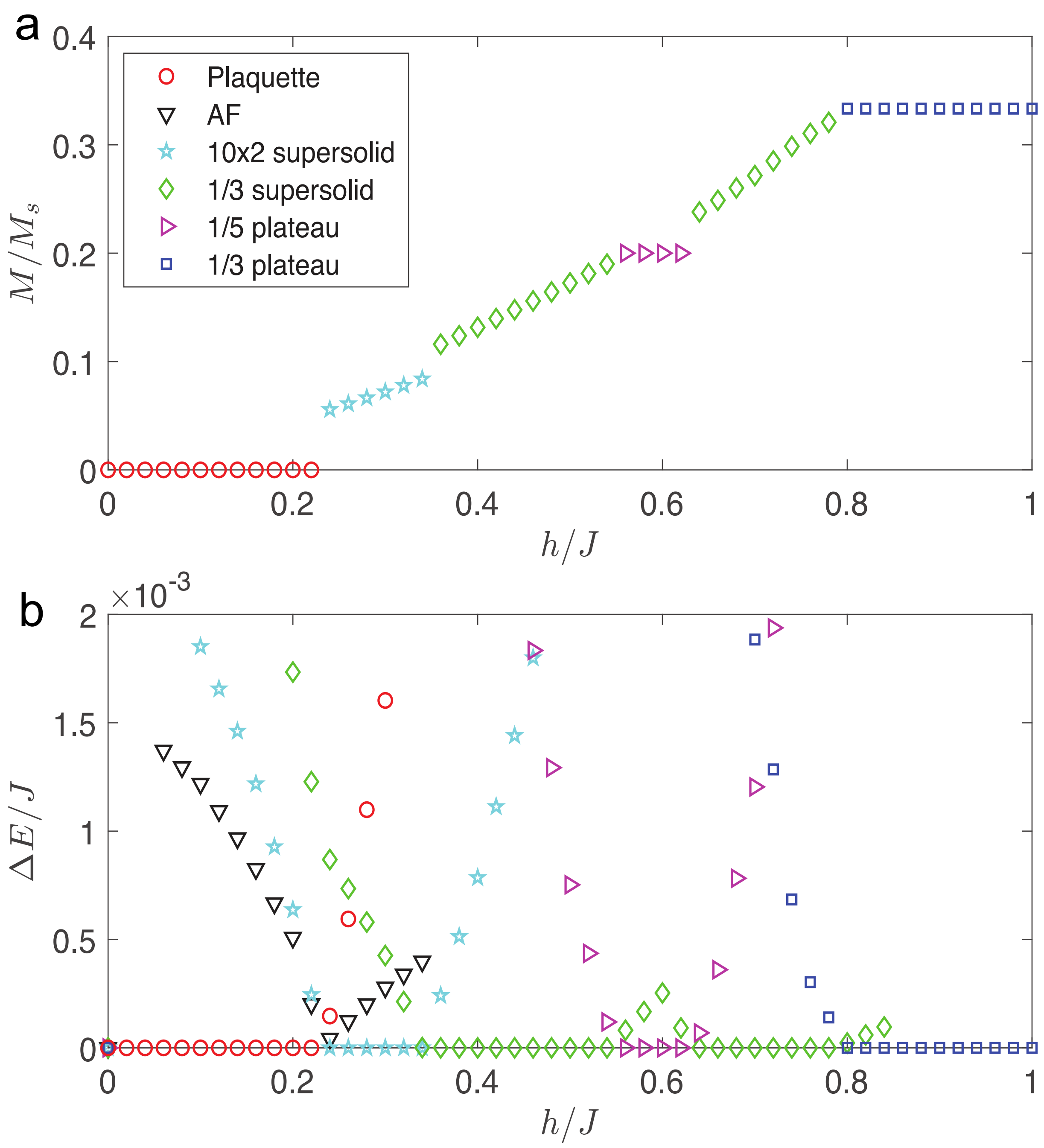

Supplementary Fig. 7: (a) iPEPS results for the magnetization curve for $J'/J = 0.68$ ($D = 8$) as a function of h/J. (b) Energy differences of the competing states with respect to the ground state as a function of h/J.

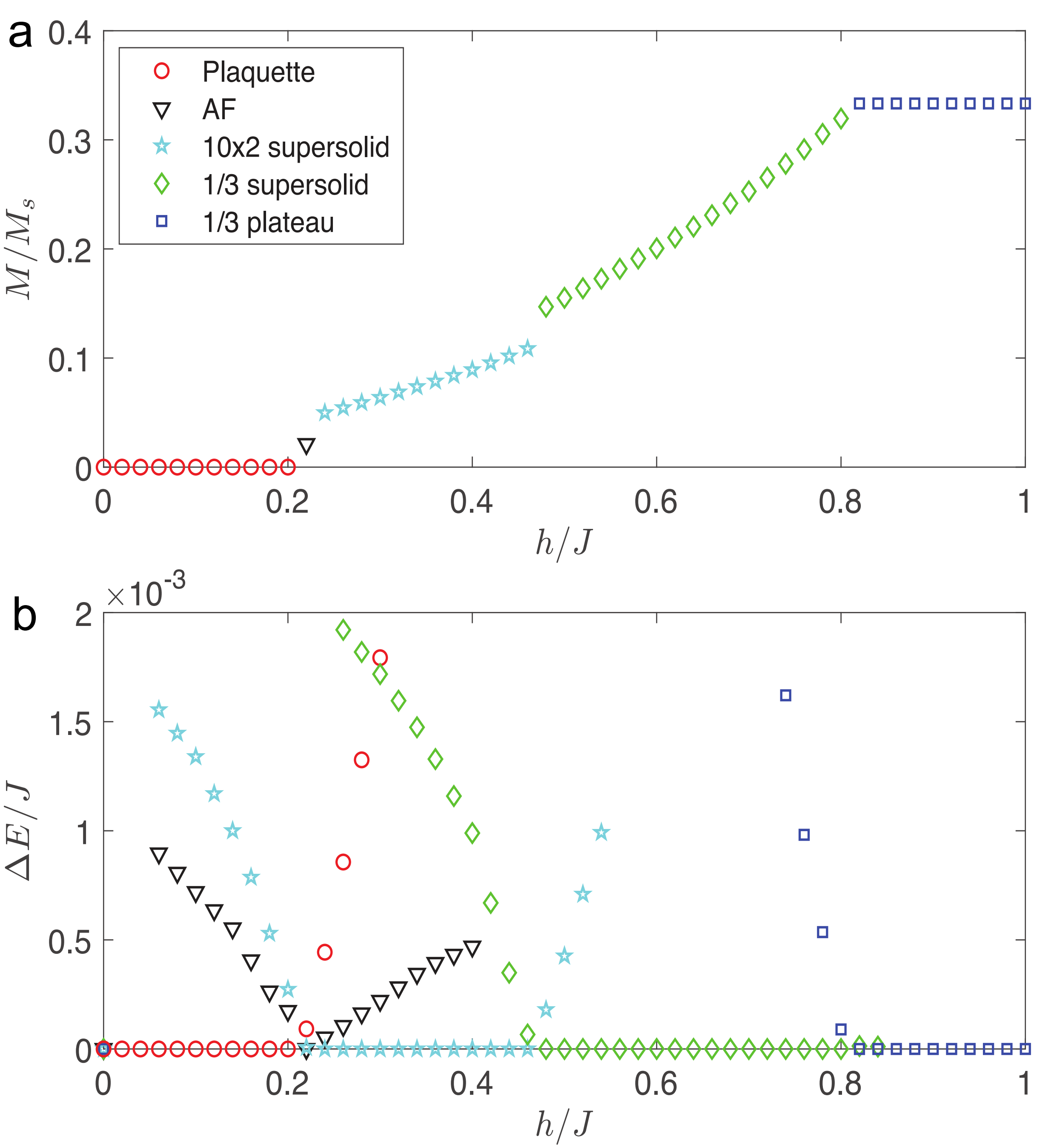

Supplementary Fig. 8: (a) iPEPS results for the magnetization curve for $J'/J = 0.7$ ($D = 8$) as a function of h/J. (b) Energy differences of the competing states with respect to the ground state as a function of h/J.

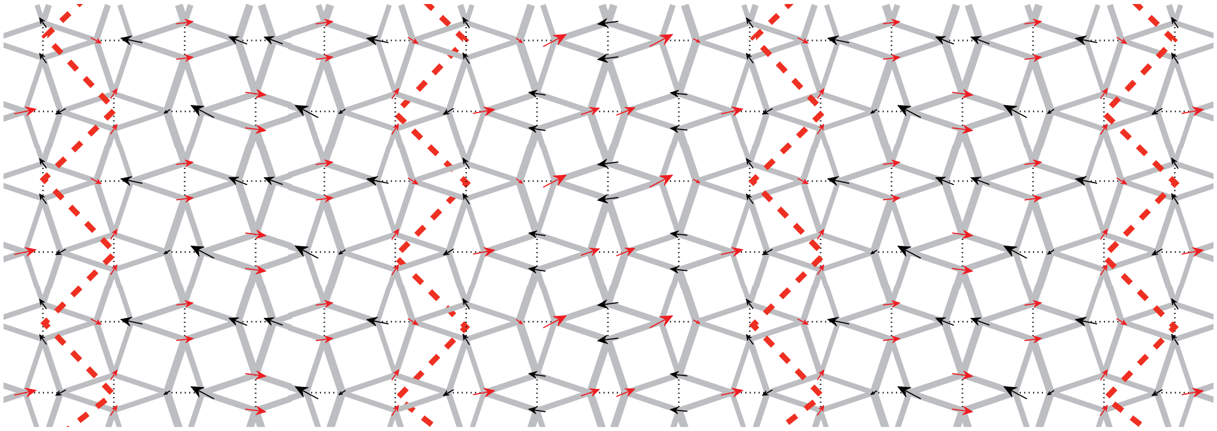

Supplementary Fig. 9: Spin structure of the 10x2 supersolid state which can be seen as a descendant of the high-P 1/5 plateau state with rotated spins. The spin component in x-direction is reversed between neighboring stripes, leading to a doubling of the unit cell size with respect to the high-P 1/5 plateau state. For a more direct comparison with the spin structure of the high-P 1/5 plateau state, the color of the spins are chosen here such that red (black) arrows show spins with a positive (negative) x-component.

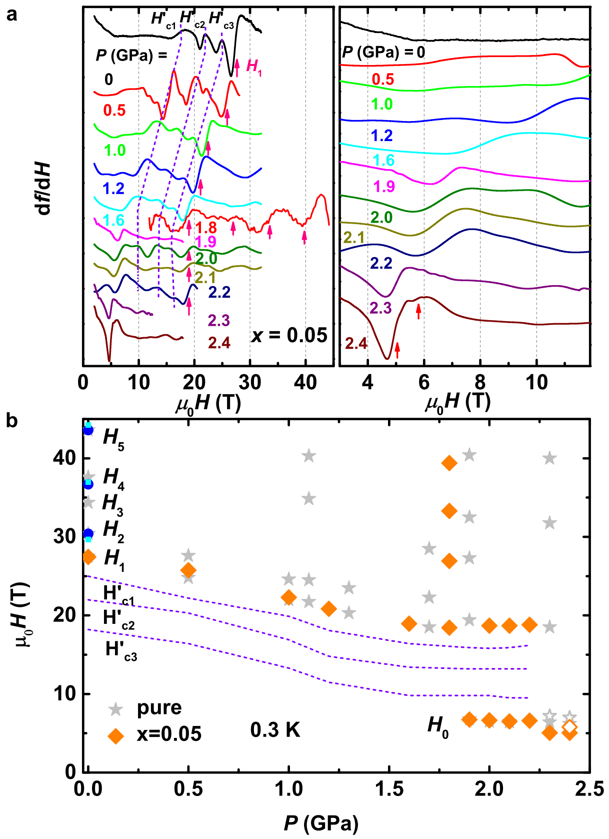

Supplementary Fig. 10: P-dependence of the magnetization plateaus and emergence of the low-field anomaly in $SrCu_{2-x}Mg_x(BO_3)_2$, for $x = 0.05$ (a)(left panel): df/dH vs H for P up to 2.4 GPa at 0.3 K. The data consist of results from multiple runs on different samples using superconducting, resistive, and hybrid magnets ($H \parallel ab$ for all measurements). The H_1 anomaly is identified for the P=0 trace and tracked to higher pressure, as indicated by red arrows. Two additional anomalies at higher fields are indicated by the red arrows for the 1.8 GPa trace. The 1/8 plateau feature is much weaker and not possible to be tracked in the doped sample. The purple dashed lines show the evolution of the H'_{c1}, H'_{c2}, and H'_{c3} anomalies (See Ref.[2] for a detailed study at $P = 0$). For the 1.8 GPa trace where data is available up to 45 T, the high-P 1/5 and 1/3 plateaus are also indicated by the red arrows (see Supplementary Fig. 3 for details). (a)(right panel): Magnified view of the low-field behavior showing the pressure dependence of the low-field anomaly. A sudden softening of the mode is seen above $P \sim 2.2$ GPa, where a splitting is also observed, as indicated by the two red arrows, similar to the $x = 0$ case. (b): $H - P$ phase diagram containing the data for $x = 0.05$ (orange) in comparison with $x = 0$ (grey). The blue and light blue symbols are defined in the same way as in Fig. 1b. The open symbols indicate the splitting of the low-field anomaly at higher P. The purple dashed lines show the pressure dependence of the H'_{c1}, H'_{c2}, and H'_{c3} anomalies.

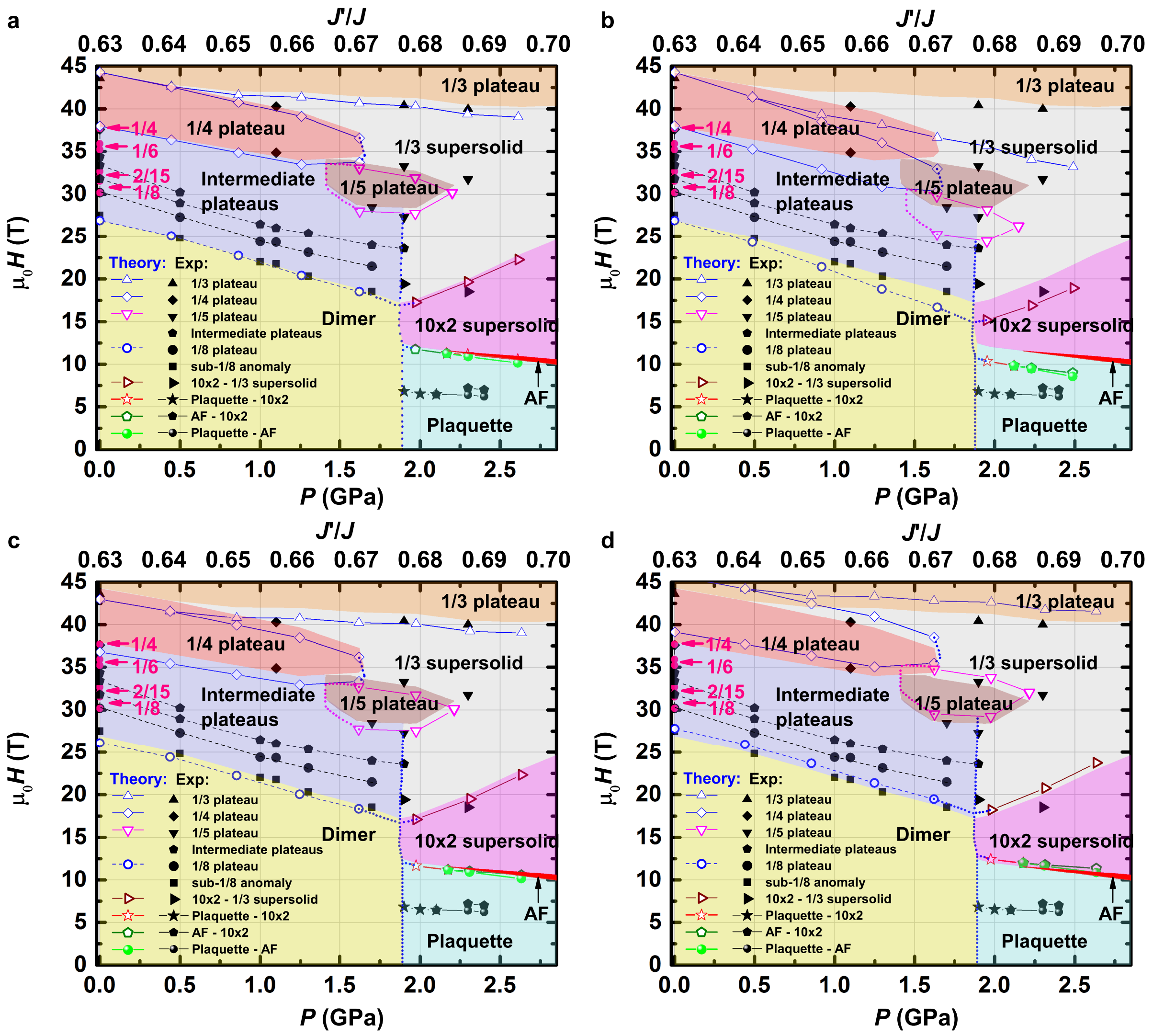

Supplementary Fig. 11: Phase diagrams obtained for different pressure dependence of $J'(P)$ and different values of $J_0 = J(P = 0)$. At $P = 0$ (ambient pressure), $J'_0/J_0 = 0.63$; At $P_c = 1.8$ GPa (critical pressure), $J'_c/J_c = 0.675$. The boundaries are marked with open symbols, while the color plots in the background represent the phase diagram shown in Fig. 3 in the main text, where $J'(P)$ decreases linearly by $\Delta_{J'} = 5\%$ between zero and critical pressure and $J_0 = 81.5$K. (a) $J_0 = 81.5$ K, $\Delta_{J'} = 7\%$. (b) $J_0 = 81.5$ K, $\Delta_{J'} = 17\%$. (c) $J_0 = 79$ K, $\Delta_{J'} = 5\%$. (d) $J_0 = 84$ K, $\Delta_{J'} = 5\%$. The results in (a) change only slightly from those in Fig. 3, while the results in (b) clearly deviate from our experimental data (black data points). In (c) and (d), the different choices of J_0 only render slight changes from the results in Fig. 3.

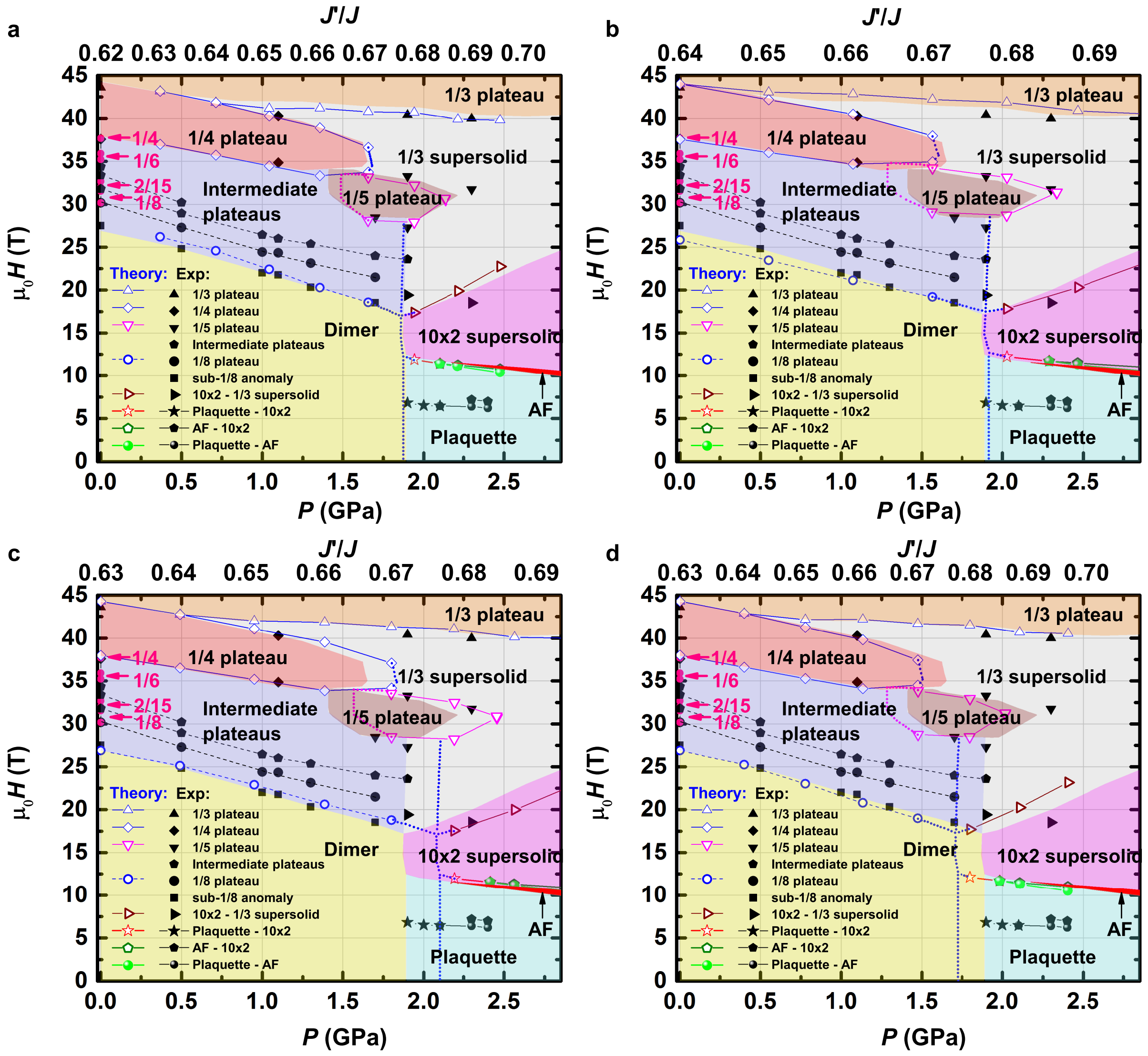

Supplementary Fig. 12: Same as in Supplementary Fig. 11 for different choices of J_0'/J_0 and J_c'/J_c, with $\Delta_{J'} = 5\%$ and $J_0 = 81.5$ K. (a) $J_0'/J_0 = 0.62$, $J_c'/J_c = 0.675$. (b) $J_0'/J_0 = 0.64$, $J_c'/J_c = 0.675$. (c) $J_0'/J_0 = 0.63$, $J_c'/J_c = 0.67$. (d) $J_0'/J_0 = 0.63$, $J_c'/J_c = 0.68$. The phase diagrams in (a-d) stay qualitatively the same and are in general agreement with the experimental data (black data points).

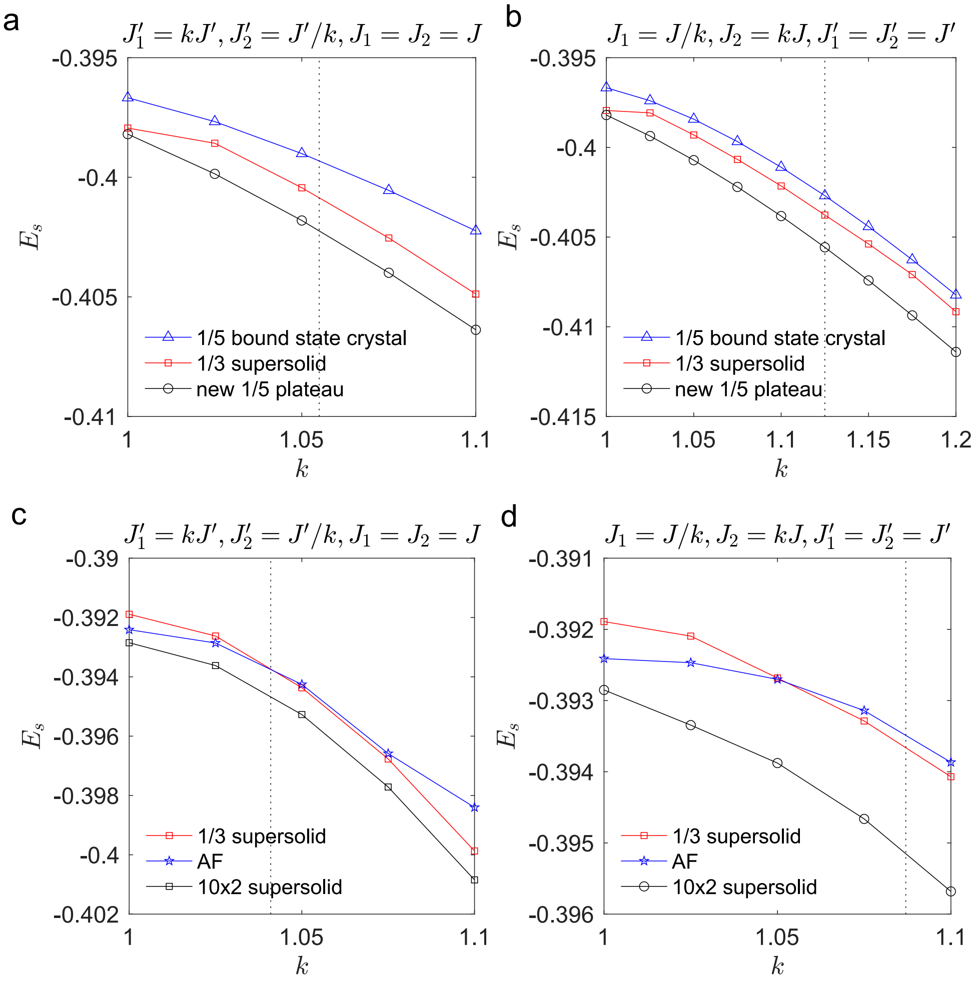

Supplementary Fig. 13: Energies of competing states for the deformed Shastry-Sutherland model from Ref. [3] with two sets of intra-dimer (J_1, J_2) and inter-dimer (J_1', J_2') couplings along different paths in the phase diagram. The parameter k controls the strength of the deformation: the larger k, the stronger the bias towards an FPP phase. The dotted vertical lines mark the transition between the empty plaquette phase (EPP) and the full plaquette phase (FPP) for $h/J = 0$. (a,b) Starting from a point in the new 1/5 plateau phase ($J'/J = 0.68, h/J = 0.6$), this phase clearly remains lowest in energy across the EPP-FPP transition, here shown for two different types of deformation. (c,d) Similar plots where the starting point ($J'/J = 0.7, h/J = 0.4$) lies in the 10x2 supersolid phase. These plots demonstrate that the new phases are relevant not only for the standard Shastry-Sutherland model, but also for the deformed model with an FPP ground state at zero field.

Main Fig. 3	$J(P)$ = 81.5 K - 5.13 K/GPa * P	$J'(P)$ = 51.3 K - 1.43 K/GPa * P
SM Fig. 11(a)	$J(P)$ = 81.5 K - 5.98 K/GPa * P	$J'(P)$ = 51.3 K - 2.00 K/GPa * P
SM Fig. 11(b)	$J(P)$ = 81.5 K - 10.2 K/GPa * P	$J'(P)$ = 51.3 K - 4.85 K/GPa * P
SM Fig. 11(c)	$J(P)$ = 79.0 K - 4.97 K/GPa * P	$J'(P)$ = 49.8 K - 1.38 K/GPa * P
SM Fig. 11(d)	$J(P)$ = 84.0 K - 5.29 K/GPa * P	$J'(P)$ = 52.9 K - 1.47 K/GPa * P
SM Fig. 12(a)	$J(P)$ = 81.5 K - 5.77 K/GPa * P	$J'(P)$ = 50.5 K - 1.40 K/GPa * P
SM Fig. 12(b)	$J(P)$ = 81.5 K - 4.49 K/GPa * P	$J'(P)$ = 52.2 K - 1.45 K/GPa * P
SM Fig. 12(c)	$J(P)$ = 81.5 K - 4.83 K/GPa * P	$J'(P)$ = 51.3 K - 1.43 K/GPa * P
SM Fig. 12(d)	$J(P)$ = 81.5 K - 5.43 K/GPa * P	$J'(P)$ = 51.3 K - 1.43 K/GPa * P

Supplementary Table 1: Linear functions of the pressure dependence of $J(P)$ and $J'(P)$ used in Fig. 3, Supplementary Figs. 11 and 12.

Supplementary Note 1

Effect of Mg dopants

At ambient pressure, the impurities introduced in $SrCu_{2-x}Mg_x(BO_3)_2$ were found to form pairs and coexist with the 2-spin dimer singlets at $H = 0$, and break down upon application of a moderate field, whereas at higher fields, the impurities interact strongly with the triplets and their bound states[2]. Four anomalies denoted by H'_0, H'_1, H'_2, H'_3 were observed at fields below the 1/8 plateau[2]. Here, H'_0 and H'_1 correspond to breakups of the impurity pairs, H'_2 corresponds to the emergence of localized bound states of triplets, and H'_3 marks the appearance of additional triplet excitations. In Supplementary Fig. 10, we show how these features evolve with the application of pressure. We note first that when measuring inside a pressure cell, the very weak anomaly of H'_0 is not observed. However, the H'_1, H'_2 and H'_3 anomalies can be tracked clearly well above 1.7 GPa, suggesting that the impurities interact with the 4-spin plaquette in a similar manner as they do with the spin dimers. This suggests that the picture of impurity-induced spin structures we established for ambient pressure[2] also applies at high pressure.

At higher fields, we still can identify the sub-1/8 anomaly at H_1 and track it to high pressure, though the more subtle 1/8 anomaly at H_2 is no longer visible in the doped sample. The low-field anomaly at H_0 also is observed (see Supplementary Fig. 10a right panel), which again splits at even higher pressure ~ 2.4 GPa. Notably, the field scale H_0 for the $x = 0.05$ sample is reduced compared to that for the pure sample. The difference emerges clearly in the doping dependence at 2.4 GPa in Supplementary Fig. 5, as well as in Fig. Supplementary 10b, where the characteristic energies for $x = 0.05$ are added to the phase diagram together with those for the pure sample. We note that for the $x = 0.02$ and $x = 0.03$ samples, we have data only up to 2.1 GPa (Supplementary Fig. 6), where the AFM phase is still negligible (Fig. 3). Based on our observations at ambient pressure[2],

it is more likely that the softening of the low energy mode (onset of the AFM phase) is a gradual change with doping. The detailed doping evolution of the softening of the low energy mode will require further exploration.

Supplementary References

[1] Onizuka, K., *et al.* 1/3 Magnetization plateau in $SrCu_2(BO_3)_2$ -stripe order of excited triplets-. *J. Phys. Soc. Jpn.* **69**, 1016 (2000).

[2] Shi, Z., *et al.* Emergent bound states and impurity pairs in chemically doped Shastry-Sutherland system. *Nat. Commun.* **10**, 2439 (2019).

[3] Boos, C., *et al.* Competition between intermediate plaquette phases in $SrCu_2(BO_3)_2$under pressure. *Phys. Rev. B* **100**, 140413(R) (2019).
